# CD4-T cell enumeration in human immunodeficiency virus (HIV)-infected patients: A laboratory performance evaluation of Muse Auto CD4/CD4% system by World Health Organization prequalification of *in vitro* diagnostics

**DOI:** 10.1371/journal.pone.0209677

**Published:** 2019-01-23

**Authors:** Ann Ceulemans, Chaymae Bouzahzah, Irena Prat, Willy Urassa, Luc Kestens

**Affiliations:** 1 Laboratory of Immunology, Department of Biomedical Sciences, Institute of Tropical Medicine, Antwerp, Belgium; 2 World Health Organization (WHO), Geneva, Switzerland; 3 Department of Biomedical Sciences, University of Antwerp, Antwerp, Belgium; Ghent University, BELGIUM

## Abstract

**Background:**

CD4 T-cell counts are still widely used to assess treatment eligibility and follow-up of HIV-infected patients. The World Health Organization (WHO) prequalification of *in vitro* diagnostics requested a manufacturer independent laboratory evaluation of the analytical performance at the Institute of Tropical Medicine (ITM) Antwerp, Belgium, of the Muse Auto CD4/CD4% system (Millipore), a new small capillary-flow cytometer dedicated to count absolute CD4-T cells and percentages in venous blood samples from HIV-infected patients.

**Methods:**

Two hundred and fifty (250) patients were recruited from the HIV outpatient clinic at ITM. Accuracy and precision of CD4 T cell counting on fresh EDTA anticoagulated venous blood samples were assessed in the laboratory on a Muse Auto CD4/CD4% system. Extensive precision analyses were performed both on fresh blood and on normal and low stabilized whole blood controls. Accuracy ((bias) was assessed by comparing results from Muse CD4/CD4% to the reference (single-platform FACSCalibur). Clinical misclassification was measured at 500, 350, 200 and 100 cells/μL thresholds.

**Results:**

Intra-assay precision was < 5%, and inter-assay was < 9%. CD4 T cell counts measured on Muse Auto CD4/CD4% System and on the reference instrument resulted in regression slopes of 0.97 for absolute counts and 1.03 for CD4 T cell percentages and a correlation coefficient of 0.99 for both. The average absolute bias as compared to the reference was negligible (4 cells/μL or 0.5%). The absolute average bias on CD4 T cell percentages was < 1%. Clinical misclassification at different CD4 T cell thresholds was small resulting in sensitivities and specificities equal or >90% at all thresholds except at 100 cells/μL (sensitivity = 87%). All samples could be analyzed as there was no repetitive rejection errors recorded.

**Conclusions:**

The Muse Auto CD4/CD4% System performed very well on fresh venous blood samples and met all WHO acceptance criteria for analytical performance of CD4 technologies.

## Introduction

In 2017, about 36.7 million people were living with HIV of which 20.7 (56.4%) were receiving antiretroviral treatment (ART) [1]. The World Health Organization (WHO) 2016 consolidated treatment guidelines recommend treatment of all HIV-infected patients irrespective of their CD4 count levels, with priority for patients with severe or advanced HIV clinical disease (WHO clinical stage 3 or 4) and adults with less than 350 CD4 T cells/μL [2]. For many years, ART initiation was based on clinical examination and absolute CD4 T-cell counts (CD4 count) and thresholds of 500, 350 and 200 CD4 T cells/μL were used to initiate ART [3]. Today, CD4 T cell counting is still considered an essential disease-specific test for health care facilities with clinical laboratories and is included in the “First-ever WHO list of essential diagnostic tests designed for the detection, diagnosis and monitoring of priority diseases such as HIV, tuberculosis, malaria, hepatitis B and C, human papillomavirus and syphilis” [4]. However, the best disease-specific marker to monitor ART in HIV patients is plasma viral load [5]. Nevertheless CD4 remains the best measurement of a patient's immune and clinical status, risk of opportunistic infections, and it is being used to support diagnostic decision-making, particularly for patients with advanced HIV disease [6]. For instance, CD4 T cell counts are used to start prophylaxis against cryptococcal infection in patients with ≤ 100 CD4 cells/μL and stop prophylaxis when patients have reached > 200 cells/μL, or to start co-trimoxazole prophylaxis to prevent HIV-related infections caused by a variety of bacterial, fungal and protozoan infections in case CD4 counts are ≤ 350 cells/μL [6,7]. As long as viral load testing is limited due to technical and financial constraints, it is expected that low-income regions will continue using CD4 counts as an alternative [8].

Ideally, laboratory evaluations of new CD4 technologies are conducted independently from the manufacturer. The WHO prequalification of *in vitro* diagnostics assessment includes an independent analytical performance evaluation of CD4 counting instruments, in particular of those meant for use in resource-limited settings [9]. During the performance evaluation, the CD4 T cell counts obtained from a new CD4 technology are compared to those from a clinical *in vitro* diagnostic (IVD) flow cytometer installed in a well-equipped accredited clinical laboratory with excellent performance in external quality assessment (EQA) schemes and operated by highly-skilled laboratory personnel [10].

Most currently used IVD flow cytometers are sophisticated large, heavy and very expensive instruments which are operated by highly skilled and dedicated personnel. They are usually used in large hospitals, are semi- or fully automated and have a high sample throughput. Fortunately; several less complicated and more affordable instruments, dedicated to CD4 T cell counting, have been developed the past two decades [11]. As a result, several portable point-of-care (POC) devices using disposable cartridges for CD4 counting have appeared on the market [12]. Those instruments are small, mobile and robust, reasonably priced and battery powered, and can be operated by health care professionals close to the patient using either finger prick or venous blood. Two of those cartridge-based POC CD4 instruments prequalified by WHO are PIMA CD4 and FACSPresto [13,14].

The gap between the large IVD instruments and the small POC devices is currently filled by a number of simplified, small flow cytometers which still require a laboratory and trained personnel but which are much easier to set up in small laboratories near the point-of-care. An example of such “intermediate” flow cytometer dedicated to CD4 counting, prequalified by WHO, which has been around for more than 20 years is the FACSCount [13,15]. In 2017, the Muse Auto CD4/CD4% System, another small intermediate flow cytometer dedicated to CD4 counting (absolute CD4 counts and CD4 T cell percentages) was evaluated at the Institute of Tropical Medicine in the context of the WHO Prequalification of in vitro diagnostic products [16,17]. This instrument is derived from the Guava Auto CD4/CD4% System but has undergone a significant hardware and software facelift. The instrument requires a laboratory, stable power supply and skilled laboratory personnel but it uses very small amounts of anticoagulated blood (10μL) and reagents, and produces a result within 30–40 minutes. Preliminary field tests of this small flow cytometer have shown very promising results [18,19]. The present study is the first manufacturer-independent study of the Muse Auto CD4/CD4% System in accordance with the WHO protocol for performance evaluation of CD4 technologies in a reference laboratory [20,21]. The main objective of the present study was to assess accuracy and precision of the Muse Auto CD4/CD4% under well-controlled laboratory conditions including the measurement of accuracy (bias) at clinical relevant CD4 thresholds: 100, 200, 350 and 500 CD4 T cells/μL.

## Material and methods

### Study population

Adult patients presenting for routine CD4 T cell enumeration at the HIV outpatient clinic of the Institute of Tropical Medicine (ITM) Antwerp, Belgium (in April-June 2017) provided a venous blood sample in 3 ml K_3_-EDTA vacutainer tubes for routine CD4 T cell counting. The same blood sample was used for CD4 T cell counting on Muse Auto CD4/CD4% System. The WHO study protocol requires collection of at least 50 blood samples from patients with <200 CD4 T cells/μL, 100 blood samples from patients with 200–500 CD4 T cells and 50 blood samples from patients with >500 CD4 T cells [21]. The study was approved by the Institutional Review Board (IRB) from ITM (1145/16). All study participants had provided a written consent that routine blood samples can be used for laboratory investigations at ITM.

### Muse Auto CD4/CD4% Systems

The laboratory evaluation was conducted with two Muse Auto CD4/CD4% Systems. The Muse CD4/CD4% System is a small capillary flow cytometer with dedicated software for CD4 T cell counting of both percentages and absolute CD4 T cell counts. The instruments needs to be installed on a steady table and requires stable external power supply. It has a large color touch screen user interface to enter patient information and reagent batch numbers. The Muse Auto CD4/CD4% reagents are a cocktail of fluorochrome-conjugated antibodies to identify all lymphocytes and CD4+ T lymphocytes. The test uses a lysing solution to lyse red blood cells prior to CD4 T cell counting. The test system comes with a Muse system check which are bead controls for system check and proficiency testing of operator (correct volume pipetting). The test requires mixing 10μL of fresh blood (<48h) with 10μL of monoclonal antibodies followed by an incubation of 15 minutes. Subsequently, 380 μL of lysing solution are added (400μL final volume) and after another incubation of 15 minutes the tube can be analyzed on the Muse instrument. The instrument reports both CD4 T cell percentages and absolute CD4 T cell counts (cells/μL).

### Analytical precision

The coefficient of variation (CV), also known as relative standard deviation (RSD), was used as a standardized measure of dispersion (precision) and was calculated as standard deviation (SD) divided by the mean of 10 repeat observation. The assessment of intra-laboratory variation of test results of Muse Auto CD4/CD4% system included instrument precision, intra-assay variation, inter-instrument variation, and inter-assay variation, using venous blood specimens. Blood samples were stratified in categories around clinically relevant CD4 thresholds: 200 (150–250), 350 (300–400) and 500 (450–550) CD4 cells/*μ*L. The CD4 category was determined by the first CD4 result of a venous blood sample measured on FACSCalibur using Trucount tubes.

Instrument precision (run-to-run) consisted of re-reading a single stained venous blood sample ten times on the same Muse Auto CD4/CD4% System. For intra-assay precision (tube-to-tube variability), staining and reading was repeated 10 times on the same blood sample and analyzed on the same Muse Auto CD4/CD4% System. For inter-instrument precision (instrument-to-instrument variability), each blood sample stained ten times and each tube was analyzed on each of the two Muse Auto CD4/CD4% Systems. The average of the obtained results was calculated for each instrument. The inter-instrument variability (CV inter-instrument) was calculated using the average result and SD of the 2 different instruments for each patient sample.

The inter-assay precision (day-to-day variability) was measured on venous blood samples analyzed within 6 hours after blood collection and reanalyzed within 48 hours after blood collection.

### Quality control and reproducibility of test results

The CD4 reference method was performed in an ISO15189:2012 certified laboratory which participates in external quality assessment programs (QASI and UK-NEQAS). The service maintenance of the CD4 reference instrument (FACSCalibur) is assured twice a year by a service engineer from BD Biosciences. The CD4 reference instrument is calibrated daily, and checked by running Multicheck Normal and Low process controls (BD Biosciences) (stabilized blood samples which can be used for several weeks). Four different batches of Normal controls and Low controls were used during the three-month duration of the study in Antwerp.

The Muse Auto CD4/CD4% System is supplied with control beads which have to be run daily as system and proficiency check prior to analyzing patients samples. Multicheck Normal and Low controls were run daily on the Muse Auto CD4/CD4% Systems to assess reproducibility of test results during the entire study period. For each control blood sample, the reported results were compared to the sample validation range provided by the manufacturer.

### Bias of Muse Auto CD4/CD4% System CD4 T cell counting results

Venous blood samples were alternatively analyzed on one of two different Muse Auto CD4/CD4% Systems each day. Absolute CD4 T cell counts and CD4 T cell percentages measured on the Muse Auto CD4/CD4% System were compared to the corresponding CD4 counting results from the FACSCalibur (BD Biosciences, San Jose, CA). CD4 counts on FACSCalibur were measured according to the manufacturer's recommendations using Multiset reagents (CD3/CD4/CD8/CD45 monoclonal antibodies) and BD Trucount tubes (both from BD Biosciences, San Jose, CA). Acquisition on FACSCalibur was automated but the individual sample analyses were done manually using CellQuest software in accordance with the accredited procedure used for routine CD4 counting in the laboratory.

The agreement between Muse Auto CD4/CD4% System results and those obtained on the reference method was assessed by Passing-Bablok regression analysis [22], Spearman Rank correlation (R) coefficient and Bland-Altman [23]. Far out negative and positive absolute bias values were identified by double-sided outlier detection according to Tukey [24]. Only when a cause, can be found for the outlier, result such as a pre-, post-, or analytical error, the outlier was removed.

The bias of Muse Auto CD4/CD4% system test results at different clinically relevant CD4 thresholds (200, 350, 500 cells/μL) and at 1000 cells/μL was calculated using Passing-Bablok regression analysis with bootstrapping of the confidence intervals of the bias at different CD4 thresholds [25]. Estimation of bias at selected threshold values was done according to CLSI guideline EP09-A3 (2013) [26].

Clinical misclassification was calculated for CD4 counts at clinically relevant CD4 thresholds of 200, 350, 500 cells/μL, using the reference method results as the “true” value for sensitivity and specificity. Inter-rater agreement was calculated with the Kappa coefficient [27].

Statistical analyses were done with MedCalc version 18.2.1 (MedCalc Software, Mariakerke, Belgium).

### Invalid results (Rejection rate)

Invalid results or sample rejection was defined as the inability of Muse Auto CD4/CD4% System to provide a CD4 results from a patient blood sample. If the analysis was aborted/rejected, a second sample was prepared and read. Upon a second rejection, the test result was recorded as sample rejection.

## Results

### Study population

In total, two hundred and fifty patients were included in this study. Clinical data was available for 236 patients, of whom 234 were HIV infected.

The median age (minimum–maximum) of the participants was 46 years (15–85) and 78% were males. Most patients (86%) were receiving antiretroviral treatment. The median (inter quartile range) CD4 T-cell count was 410 (252–582) cells/μL; 52 had CD4 < 200 cells/μL (median 129 (65–180) cells/μL), 113 had between 200–500 CD4 T cells/μL (median 386 (312–437) cells/μL), 85 had > 500 CD4 T cells/μL (median 753 (581–948) cells/μL). None of the patients presented with malaria; 4 patients had clinical features suggestive of pulmonary tuberculosis. From 14 patients clinical data was not available.

### Analytical precision

The mean instrument precision for absolute CD4 T cell counts and CD4 T cell percentages in venous blood varied between 2.5 and 5.5% depending on the CD4 category. The intra-assay CV varied from 2.8 to 4.6%. The inter-assay CV varied from 3.5 to 8.1%. The inter-instrument CV varied from 2.5 to 4.4%. The individual CV for the different CD4 categories and for each type of precision assay are presented in Table 1.

**Table 1:** Precision of CD4 T cells counts and percentages at different categories of CD4 T cells/μL.

	Instrument		Intra-assay		Inter-assay		Inter-instrument	
	Precision (CV)		Precision (CV)		Precision (CV)		Precision (CV)	
**CD4 category**	CD4	CD4%	CD4	CD4%	CD4	CD4%	CD4	CD4%
**(cells/μL)**	N = 5	N = 5	N = 4	N = 4	N = 4	N = 4	N = 3	N = 3
**150–250**	5.5	5.1	4.5	4.4	6.9	4.0	4.4	3.4
**300–400**	3.6	3.6	4.6	3.7	8.1	3.5	3.6	3.4
**450–550**	3.4	2.5	4.0	2.8	7.4	4.6	3.5	2.5

CV, coefficient of variation (%); CD4, CD4 T cells/μL; CD4%, CD4 T cell percentage; N, number of different blood samples used per CD4 category.

### Quality control of Muse Auto CD4/CD4% System and reproducibility of results using normal and low CD4 controls

Four different Multicheck batches (lots) of Normal and Low controls were used over the entire laboratory evaluation including 36 consecutive measurements of a Normal and Low CD4 count control over the entire study duration of 3 months. The Low controls were stabilized blood samples with CD4 counts between 100–200 CD4 T cell counts/μL. The Normal controls were stabilized blood samples with CD4 T cell counts between 600–1000 CD4 T cells/μL. The average CV for absolute CD4 T cell counts over the entire study duration was <5% for Normal CD4 count controls and <8% for Low CD4 T cell count controls. The overall CV for CD4 percentages was <2% for Normal controls and <6% for Low controls.

### Bias of Muse Auto CD4/CD4% System

**Fig 1:**
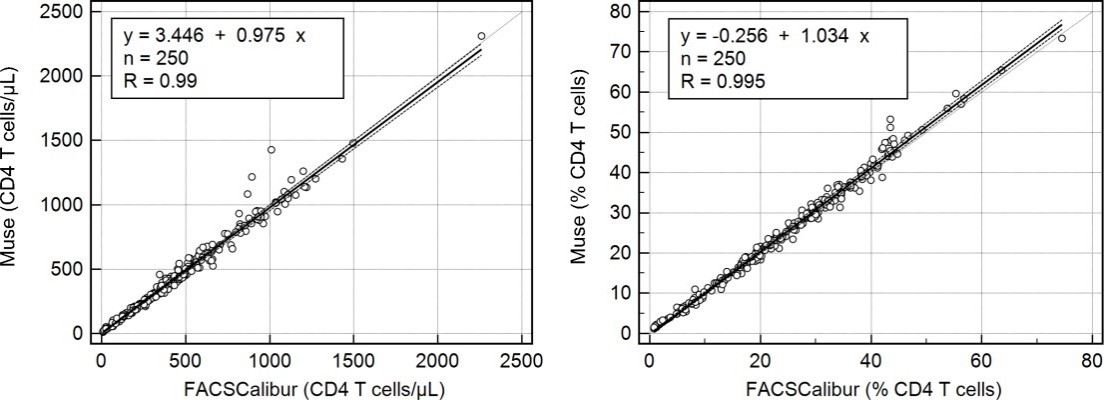
Passing-Bablok regression for absolute CD4 T cell counts (left) and CD4 T cell percentages (right).

**Fig 2:**
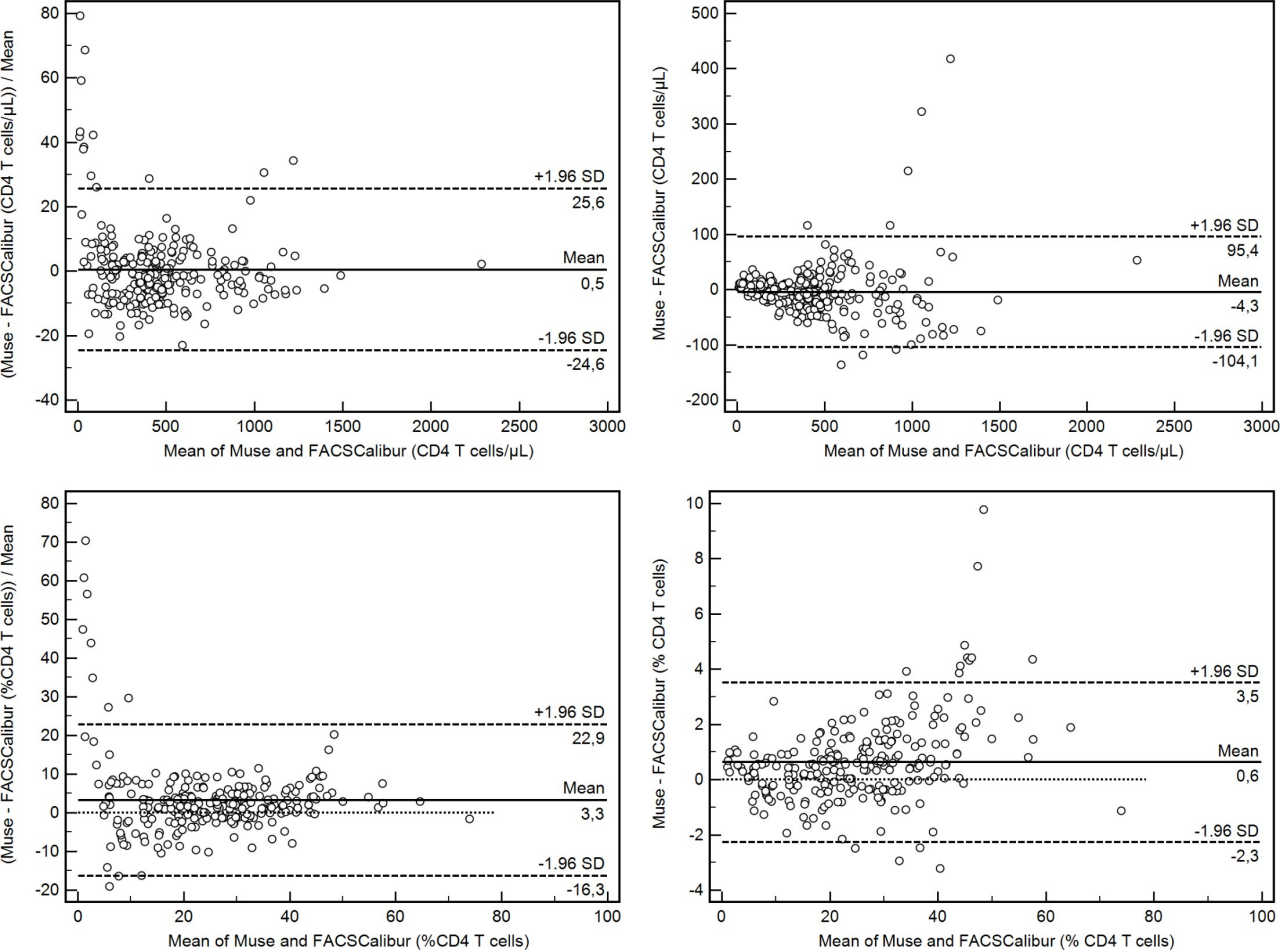
Bland-Altman analysis for absolute CD4 T cells counts and CD4 T cell percentages.

Agreement between Muse Auto CD4/CD4% System and the reference method is shown in Fig 1 (Passing-Bablok), Fig 2 (Bland-Altman), Table 2 (correlation and bias) and Table 3 (bias at clinically relevant CD4 thresholds). Fig 1 shows the Passing-Bablok regression between the results of Muse Auto CD4/CD4% System and those of the reference technology for both absolute CD4 T cell counts and percentages. The slopes of the regression equations ranged from 0.97–1.04. The spearman rank correlation coefficients were >0.99.

**Table 2:** Performance of Muse Auto CD4/CD4% System compared to FACSCalibur (reference).

	CD4 T cells/μL	% CD4 T cells
**Regression**^a^	y = 3.45+0.975x	y = 0.26+1.03x
(95% CI of slope)	(0.96;0.99)	(1.02;1.05)
Spearman Rank Correlation R	0.99	0.99
**Mean absolute bias (LOA)**^b^	-4.3 (-104;+95) cells/μL	0.6 (-2.3;+3.5)%
**Mean relative bias (LOA)**^c^		
All results	0.5 (-25;+26)%	3.3 (-16;+23)%
For results > 100 CD4 T cells/μL	-1.6 (-17;+14)%	1.8 (-8.3;12)%

CI, confidence intervals; LOA, limits of agreement (95% CI of the mean difference).

^a^Passing-Bablok regression

^b^Bland-Altman analysis on absolute differences

^c^Bland-Altman analysis on relative differences.

**Table 3:** Estimation of bias of Muse Auto CD4/CD4% System at different clinically relevant CD4 T cell thresholds.

Bias Estimation at X^a^	Absolute bias	Relative bias
	cells/μL (95% CI)	% (95% CI)
**100 CD4 T cells/μL**	0.99 (-4.2;+4.4)	0.98 (-4,3;+4,3)
**200 CD4 T cells/μL**	-1.5 (-5.2;+0.9)	-0.74 (-2.6;+0.43)
**350 CD4 T cells/μL**	-5.2 (-8.8;-3.0)	-1.5 (-2.6;+0.43)
**500 CD4 T cells/μL**	-8.8 (-14;-5.2)	-1.8 (-2.8;-1.0)
**1000 CD4 T cells/μL**	-21 (-33;-9.6)	-2.1 (-3.4;-0.97)

^a^Absolute and relative bias estimation at different CD4 T cell thresholds was calculated using Passing-Bablok regression.

Fitted line equation, the number of analysed samples (n) and Spearman rank correlation are depicted in the legends.

Absolute bias on absolute CD4 T cells counts (top left), absolute bias on CD4 T cells percentages (top right). Relative bias on absolute CD4 T cells counts (bottom left), relative bias on CD4 T cells percentages (bottom right).

The absolute and relative bias was calculated for absolute CD4 T cell counts and for CD4 T cell percentages (Table 2).

As the relative bias can be large at low CD4 T cell counts, without being meaningfull, the relative bias was recacluated for patients with > 100 CD4 T cell counts resulting in a smaller, and thus better limits of agreement (Table 2). As the bias is inconsistent over the entire range of CD4 T cells counts, it was calculated at clinically relevant CD4 thresholds (Table 3). The relative bias on absolute CD4 T cells count varried slightly per threshold from <-0.74% at 200 CD4 T cells/μL to—2.1% at 1000 CD4 T cells/μL representing less than -2 cells on average at the 200 threshold and -21 cells on average at 1000 CD4 T cells.

### Clinical misclassification

Misclassification analysis is shown in Table 4. Kappa coefficient ranged from 0.90 to 0.96 for the different thresholds (200, 350 and 500 cells/μL) indicating a good agreement between the two systems. Sensitivity and specificity were in all cases equal or higher than 90%. A misclassification means that the clinician will not start ART at the expected time point.

**Table 4:** Clinical misclassification of Muse Auto CD4/CD4% system at strict thresholds of 100, 200, 350 and 500 CD4 T cells/μL.

Decision Threshold	< 100 cells/μL	< 200 cells/μL	< 350 cells/μL	< 500 cells/μL
**N**	250	250	250	250
**N Disease (%)**	21 (8,4%)	47 (18.8%)	88 (35.2%)	162 (64.8%)
**Kappa**	0.89	0.90	0.91	0.96
**Sensitivity (%)**	86	90	97	98
**Specificity (%)**	99.6	98	95	98

N, number of individuals; N Disease (%), number and percentage of patients with absolute CD4 T cell counts below decision threshold; Kappa, Inter-rater agreement Kappa

### Invalid results (Rejection rate)

During the study, the rejection rate was 0/250 (0%). All blood samples could be analyzed on Muse Auto CD4/CD4% System and all results were included in the study.

### Operators’ feed-back

Technicians found the Muse Auto CD4/CD4% instrument easy to use after a short 4-hour training.

The keypad on the screen used to enter details on the sample, user, batch numbers etc was considered a bit small and technicians with larger fingers (e.g larger male users) had trouble pressing the correct keys. The small blood volume (10μL) and reagent volume (10μL) required to perform the test was a concern at the start of the study but, as supported by the results, that did not have any impact on the precision and accuracy of the measurements. The system check at start-up which includes an operator proficiency check is considered an asset as it will assure accurate pipetting. The instrument has no sample loader and samples are analyzed on the instrument manually. The manufacturer recommends an extra instrument cleaning step after 20 measurements to prevent needle clogging. Since preparation of 20 samples takes about 45min to 1 hour and the analysis of 20 samples on the instrument take another hour, 80 samples can be analyzed on an 8h working day per technician and per instrument. In theory, a first result can be generated after 1 hour.

The Muse CD4/CD4% instruments are much smaller than conventional large IVD flow cytometers, easier to move around (mobility) and simple to operate (much less training required).

The system reported warnings but the frequency was low (<5%). Most of the warnings were related to a concern about correct automatic gating and required visual inspection and adjustment of the gate. Setting the instrument takes about 15 min (complete system clean + system check) before blood samples can be analyzed. Technicians consistently implemented the wash procedure which can optionally be skipped although it is not recommended to do so. At the end of a day, the flow cell needs to be cleaned by a thorough cleaning procedure (designated “Extreme Clean Procedure”) which takes about 15 min. The technicians did not experience any clogging of the needle during the study. The capillary needle of the flow cell in the instrument is very delicate and replacement should be done very carefully. Technicians never had to replace the flow cell during the study.

## Discussion

The laboratory evaluation of the Muse Auto CD4/CD4% system was conducted in accordance with the WHO prequalification evaluation protocol to verify whether this system met the WHO acceptance criteria for CD4 technologies [20]. Those acceptance criteria have been established to assure that CD4 technologies are sufficiently reliable and produce good results. Although the entire prequalification process verifies many other aspects including reagent stability, instrument robustness, the laboratory performance evaluation is conducted to verify accuracy (proximity to the true value) and precision (reproducibility). Precision of Muse Auto CD4/CD4% determined on fresh blood samples was found to be good and well below the maximum allowable CV of 10%, for >200 CD4 T cells/μL or below15% for <200 CD4 T cells/μL. To assess the day-to day reproducibility of results, fresh blood samples cannot be used for longer than 48h to be in accordance with the instructions for use provided by the manufacturer. Therefore, stabilized Normal and Low CD4 count whole blood controls were used to assess day-to-day reproducibility on the Muse Auto CD4/CD4% system. The average CV for absolute CD4 counts over the entire study duration was <5% for Normal CD4 count controls and <8% for Low CD4 count controls which is well within the WHO acceptance limits.

Accuracy (bias) was assessed by comparing results of the Muse Auto CD4/CD4% to the results obtained by an established reference method/instrument. This is the easiest way to assess accuracy of a new CD4 technology as there are no international reference blood samples to assess the bias Alternatively, the bias can be assessed by comparing the results a CD4 technology to the consensus value of an external quality assessment program (EQA). The consensus value is defined as the average CD4 result of all laboratories participating laboratories in the EQA program and the difference between the individual result and the consensus value is a good estimated of the bias. During the present study, the average difference (bias) between Muse Auto CD4/CD4% and the FACSCalibur results were negligible (0.5% or 4 cells), and well within the maximum allowable relative bias of 10%. This small bias is confirmed by the regression analysis with a slope close to 1 (0.97), and a correlation of 0.99. There was a very slight tendency towards larger underestimation at higher CD4 T cell counts as the relative bias increased from -0.7% to -2.1% between 200 and 1000 CD4 T cell counts (Table 3).

In case the Muse Auto CD4/CD4% would be used for taking decisions with regard to treatment initiation, the present study found that sensitivity at 100 CD4 T cells/μL was 86% and ≥90% at the higher thresholds with a specificity of ≥95% indicating that the number of misclassified patients would be relatively low (Table 4).

The instrument uses small volumes of blood and reagent (10μL each). This could be considered as a concern as pipetting of such small volumes may introduce larger errors if pipets are not properly calibrated and technicians not properly trained. However, this was not found to be an issue during this study as the precision studies produced very good results. The obligatory pipetting proficiency test (pass/fail) prior to running blood samples is certainly helping to assure good pipetting practice.

Until today, two field studies of the Muse Auto CD4/CD4% have been published, both by the same group [18,19]. One study, conducted in the Central African Republic, also looked at analytical performance, and although the number of enrolled patients was smaller than requested by WHO prequalification, and a FACSCount was used as predicate instrument, both precision and bias estimates were within the WHO acceptance criteria. The second study assessed whether the Muse could be used by trained lay providers. Results were found to be less precise and accurate, but the study demonstrated that the instrument can be used to decentralize CD4 counting to health community centers [19].

The observation that the Muse Auto CD4/CD4% is small but precise and accurate instrument is encouraging and instruments like the Muse could be used to monitor HIV disease and to screen patients for the risk of opportunistic infections. Even when CD4 T cell counting will lose importance in monitoring ART, instruments like Muse Auto CD4/CD4% are still considered to be as an essential disease-specific IVD tool for health care facilities with clinical laboratories [4].

The limitation of the present study is that the analytical performance was conducted in a Western accredited laboratory with well-trained technicians. This study did not assess information on robustness and temperature and humidity fluctuations common to rural areas. Although our study validated the analytical performance characteristics of the instrument, more field studies are required to assess the performance of the Muse Auto CD4/CD4% in real-life conditions typical of rural settings.

In conclusion, the Muse Auto CD4/CD4% system showed a very good analytical performance on fresh venous blood samples. The instrument was considered easy to use, with minimal training and infrastructure required, making it suitable for decentralization of CD4 T cell counting in rural resource-limited settings with a small laboratory, trained technicians and stable power supply.

## Supporting information

S1 Database Raw data database WHO Muse Auto CD4 study.xlsx. (XLSX)
